# Biogenic Synthesis of Copper and Silver Nanoparticles Using Green Alga *Botryococcus braunii* and Its Antimicrobial Activity

**DOI:** 10.1155/2018/7879403

**Published:** 2018-10-21

**Authors:** Anju Arya, Khushbu Gupta, Tejpal Singh Chundawat, Dipti Vaya

**Affiliations:** ^1^Department of Applied Sciences, The North Cap University, Sector 23-A, Gurugram 122017, Haryana, India; ^2^Department of Chemistry, Amity University, Gurugram, Haryana, India

## Abstract

The spread of infectious diseases and the increase in the drug resistance among microbes has forced the researchers to synthesize biologically active nanoparticles. Improvement of the ecofriendly procedure for the synthesis of nanoparticles is growing day-by-day in the field of nanobiotechnology. In the present study, we use the extract of green alga *Botryococcus braunii* for the synthesis of copper and silver nanoparticles. The characterization of copper and silver nanoparticles was carried out by using UV-Vis spectroscopy, Fourier transform infrared spectroscopy (FTIR), X-ray diffraction (XRD), and scanning electron spectroscopy (SEM). FTIR measurements showed all functional groups having control over reduction and stabilization of the nanoparticles. The X-ray diffraction pattern revealed that the particles were crystalline in nature with a face-centred cubic (FCC) geometry. SEM micrographs have shown the morphology of biogenically synthesized metal nanoparticles. Furthermore, these biosynthesized nanoparticles were found to be highly toxic against two Gram-negative bacterial strains *Pseudomonas aeruginosa* (MTCC 441) *and Escherichia coli* (MTCC 442), two Gram-positive bacterial strains *Klebsiella pneumoniae* (MTCC 109) and *Staphylococcus aureus* (MTCC 96), and a fungal strain *Fusarium oxysporum* (MTCC 2087). The zone of inhibition was measured by the agar well plate method, and furthermore, minimum inhibitory concentration was determined by the broth dilution assay.

## 1. Introduction

Nowadays, nanotechnology is a notable efficacious field for research work [1]. Nanotechnology is the branch of science and technology, which deals with the production of substances in size less than 100 nm scale as nanoparticles [2]. Among other nanoparticles, metal nanoparticles have raised attention over the last few decades because they have larger surface area per weight or to volume and many characteristics; biological, thermal, chemical, dielectric, electrical, physical, mechanical, electronic, magnetic, and optical properties make them attractive tools for research work [3]. Therefore, nanoparticles are studied as the building blocks of the next generation of technology with applications in different industrial areas. In particular, metal and metal oxide nanoparticles are receiving optimum attention in a large variety of applications [4]. Nanoparticles of transition metals are an important class of semiconductors, which have applications in magnetic storage media, solar energy transformation electronics, gas sensors, and catalysis [5–7]. The nanoparticles play up the most important role in different disciplines such as health care, screening and medicines, drug delivery system, antisense, tissue biotechnology, cosmetics, applications of gene engineering, and in many other fields [8, 9].

Currently biological species like algae are in great use for the nanoparticles synthesis. Both live and dead biomass of algae is used for the biogenic synthesis of nanoparticles that is why they are called as bionanofactories. Algae have good metal uptake capacity, and thus, the biological method with the use of algae is cost-effective and ecofriendly [10]. Algae contain large amount of the reducing agent which reduces metal salts to their respective metal nanoparticles without any hazardous by-products. Aqueous extract of algae contains secondary metabolites such as polysaccharide, proteins, tannins, and steroids as bioactive molecules [11–13].

Synthesized silver and copper nanoparticles also showed antimicrobial activity [14–16]. The nanoparticles have effective antibacterial and antifungal property due to their large surface area, which allow them a better contact with microbes [17]. The nanoparticles can disorder the helical structure of DNA by cross-linking within the nucleic acids stands, and they also disrupt the biochemical processes [18].

Nanoparticles with antimicrobial activity are very advantageous in reducing acute toxicity, lowering cost, and overcoming resistance as compared with other prevalent antibiotics [19, 20]. Antibiotics can sustain for long run in the form of nanoparticles than any other large or small molecules [21].

The previous findings of research on biogenic synthesis of silver and copper nanoparticles using green algae have been unexplored; there are few literatures found on silver nanoparticles [22–26] but on copper nanoparticles less and none using green alga *Botryococcus braunii*. The present study has shown that the use of green alga *Botryococcus braunii* as biofactory for synthesis of silver and copper nanoparticles and their antimicrobial activities. To best of our knowledge and understanding, there is no report on synthesis of silver and copper nanoparticles using green alga *Botryococcus braunii*. The synthesized nanoparticles were characterized by different techniques used such as UV-visible spectroscopy, scanning electron microscopy, X-ray diffraction, and Fourier infrared spectroscopy. Furthermore, antimicrobial activity against bacterial and fungal species was also exhibited by biogenically synthesized silver and copper nanoparticles.

## 2. Materials and Methods

### 2.1. Materials

Green alga *Botryococcus braunii* was collected from Udaisagar Lake Udaipur (Rajasthan, India). The reagents agar-agar, copper acetate, and silver nitrate are of analytical grade and were purchased from Sigma-Aldrich. Bacterial strains like *Pseudomonas aeruginosa* (MTCC 441), *Escherichia coli* (MTCC 442), *Klebsiella pneumoniae* (MTCC 109), and *Staphylococcus aureus* (MTCC 96) and a fungal strain *Fusarium oxysporum* (MTCC 2087) were purchased from the microbial type collection, Chandigarh, India.

### 2.2. Methods

#### 2.2.1. Isolation and Culturing of Green Alga and Algal Extract Preparation

Green alga was isolated by the serial dilution method and grown on the Chu-13 nutrient medium solidified by 1.5% agar-agar. The algal colonies appearing after three weeks of incubation were isolated and inoculated into the liquid medium. For experiments, alga was grown for algal biomass in a incubator at 27 ± 1°C, 1.2 ± 0.2 Klux light intensity, and 16 : 8 hrs light: dark cycle in the nutrient medium. Grown algal biomass was centrifuged, shade dried, and taken 5 g of algal biomass in a 250 ml Erlenmeyer flask along with 100 ml of distilled water. Mixture was autoclaved for 15 min and filtered hot through the Whatman No. 1 filter paper. The filtered extract was centrifuged, and the supernatant was used as the reducing agent for preparing metal nanoparticles. Prepared algal extract was kept at 4°C in a refrigerator for future use.

#### 2.2.2. Synthesis of Metal Nanoparticles

*(1) Biogenic Synthesis of Copper Nanoparticles*. 5 ml algal extract was added dropwise into 50 ml of 1 mM aqueous copper acetate in a 100 ml Erlenmeyer flask with vigorous stirring at 100°C for 24 h. Simultaneously, a positive control of copper acetate aqueous solution and algal extract and a negative control containing only copper acetate aqueous solution were maintained under the same conditions. In the positive control within three hours, the light sky blue solution changed to dark brown. It was the indication of formation of copper nanoparticles, but in the negative control, the colour remains unchanged. The progress of the process was regularly monitored by observing colour change and recording UV-visible spectrum. After the completion of the process, the above reaction mixture was centrifuged for 15 min and the obtained material was subsequently redispersed and washed with deionised water to remove debris and any uncoordinated biomolecules. This process of separation and washing was carried out thrice to certain severance of copper nanoparticles. These copper nanoparticles were dried in a oven for further characterization.

*(2) Biogenic Synthesis of Silver Nanoparticles*. 5 ml algal extract mixed with 45 ml and 1 mM silver nitrate aqueous solution in a 100 ml Erlenmeyer flask were put on magnetic stirrer at room temperature for 3 h. Simultaneously, a positive control of silver nitrate aqueous solution and algal extract and a negative control containing only silver nitrate aqueous solution were maintained under the same conditions. The progress of the process was regularly monitored by observing colour change and recording UV-visible spectrum. In the positive control, the initial light pale yellow solution turned to reddish brown, indicating formation of silver nanoparticles, but in the negative control, no change in colour was found. After the reaction reached saturation, the solution was centrifuged for 20 min, and the obtained pellet was washed with deionised water to remove impurities. This process of centrifugation and washing was carried out thrice to get a better separation of nanoparticles. The obtained silver nanoparticles were oven-dried at 55°C for 5 h.

#### 2.2.3. Characterization of Metal Nanoparticles

The bioreduction of metal ions in solution was monitored using a UV-Vis NIR spectrophotometer (Model no. Cary series) taking a spectra from 200 to 800 nm for each sample against distilled water as blank. The dried powders of copper and silver nanoparticles were used for characterizations. FTIR analyses were carried out on FTIR (PerkinElmer) in the range of 4000–450 cm^−1^ using dried powder of metal nanoparticles. Samples for analysis were prepared at ambient conditions and mixed with KBr, and X-ray diffraction measurements were carried out on the Philips Xpert pro XRD system (DY 1650). The shape and size of copper and silver nanoparticles were analyzed by using scanning electron microscopy (SEM) images obtained with the help of scanning electron microscope (Model-FEI Quanta 200 SEM).

#### 2.2.4. Antimicrobial Activity of Synthesized Metal Nanoparticles

Antimicrobial activity of green-synthesized nanoparticles was evaluated by the agar well plate method, and minimum inhibitory concentration was determined by microbroth dilution assay. Two Gram-negative bacterial strains *Pseudomonas aeruginosa* (MTCC 441) *and Escherichia coli* (MTCC 442), two Gram-positive bacterial strains *Klebsiella pneumoniae* (MTCC 109) and *Staphylococcus aureus* (MTCC 96), and a fungal strain *Fusarium oxysporum* (MTCC 2087) were used in the investigation. Bacterial cultures were maintained in Petri plates containing the nutrient agar (NA) medium at 37°C, and the fungus *Fusarium oxysporum* was maintained in potato dextrose agar at 25°C. All the cultures were subcultured on regular basis and stored at 4°C.

#### 2.2.5. Agar Well Plate Assay

The agar well diffusion method was used to evaluate the antimicrobial activity of synthesized nanoparticles. The nutrient agar containing the microbial inoculum was spread all over the Petri plate. Allow it to cool for some time, and then a well of diameter 8–10 mm was punched aseptically with a sterile cock borer or a tip. 20–40 ml of 1000 *µ*g/ml concentration of nanoparticles was added into the well, and then the agar plates were incubated under sterile conditions depending upon the test microorganism. The nanoparticles diffuse into the agar media and inhibit the growth of microbes. Then, the antimicrobial activity of the nanoparticles was detected by the appearance of the inhibition zone around the agar well. Zone was measured with the help of a transparent ruler from one edge to the other edge of the clear area.

#### 2.2.6. Broth Dilution Method

For this method, all synthesized nanoparticles were diluted to obtain a stock solution having 2000 *µ*g/ml concentration. For the primary screening, these synthesized nanoparticles were diluted obtaining 1000 *µ*g/ml, 500 *µ*g/ml, and 250 *µ*g/ml concentrations. The nanoparticles found active against microbes in the primary screening were further diluted in the secondary screening to obtain 200 *µ*g/ml, 100 *µ*g/ml, 50 *µ*g/ml, 25 *µ*g/ml, 12.5 *µ*g/ml, and 6.250 *µ*g/ml concentrations. Each synthesized nanoparticles were diluted using DMSO as a diluent. The highest dilution showing at least 99% inhibition zone is taken as MIC. The MIC value is very much affected by the size of the inoculums. The test mixture should contain approximately 10^8^ number of organisms/ml.

## 3. Results and Discussion

**Figure 1:**
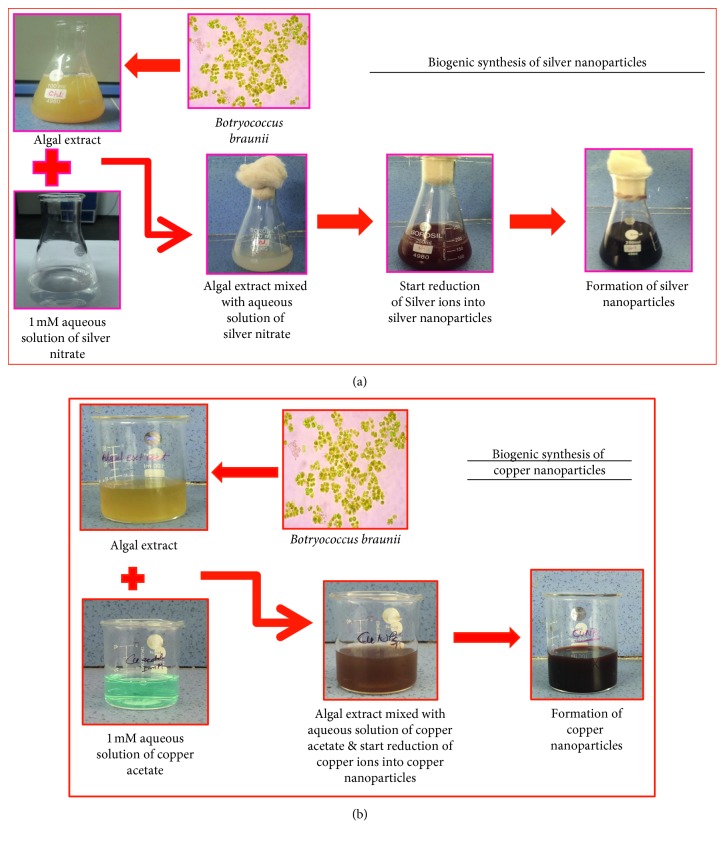
(a) Biogenic synthesis of silver nanoparticles; (b) biogenic synthesis of copper nanoparticles.

In this research, we have used green alga *Botryococcus braunii* for biogenic synthesis of copper and silver nanoparticles. Synthesized copper and silver nanoparticles were examined for antimicrobial activity against bacterial and fungal species. The alga-mediated biogenic synthesis of both nanoparticles as copper and silver is given in Figure 1. Synthesized metal nanoparticles were initially confirmed by visual observation as change in colour. Furthermore, UV-visible spectra have been regularly used for characterization of biogenically synthesized nanoparticles. Surface plasmon resonance phenomenon provides a convenient signature to indicate the formation of copper and silver nanoparticles by change in colour in the reaction mixture [27]. Aqueous extract of green alga *B. braunii* was added into aqueous solution of copper acetate and silver nitrate for copper and silver nanoparticles, respectively. The formation of copper and silver nanoparticles was characterized by colour change from light sky blue to dark brown and light pale yellow to reddish brown through visual observation means, indicating the reduction of copper and silver ions into their respective nanoparticles [27, 28]. In this reduction process, metal nanoparticles scatter and absorb light at a certain wavelength due to the resonant excitations of charge density at the interface between a conductor and an insulator, the phenomena called surface plasmon resonance [29].

### 3.1. UV-Vis Spectroscopy

**Figure 2:**
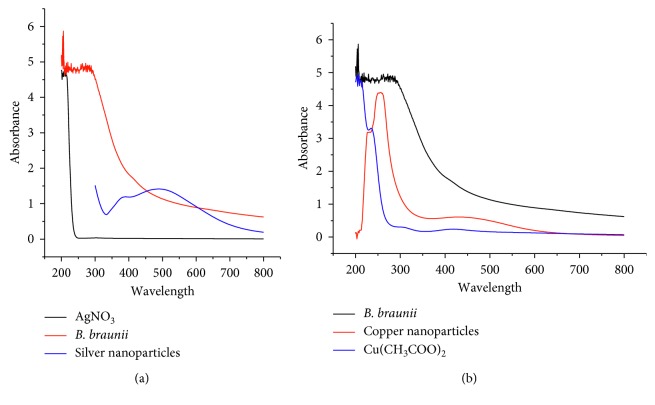
UV-Vis spectra of (a) silver nanoparticles and (b) copper nanoparticles.

UV-visible spectrum of silver and copper nanoparticles synthesized by *B. braunii* exhibited the absorption peaks around 490 nm and 258 nm, respectively (Figure 2). The synthesized silver nanoparticles show absorbance at 490 nm according to previous findings [22]. In case of copper nanoparticles, two peaks were found: first one at 258 nm is attributed to formation of copper nanoparticles [14, 30, 31], and second one at 460 nm ascribed to the presence of the oxide shell around nanoparticles [32]. These peaks have already been recorded for various metal nanoparticles which ranged from 2 to 100 nm in size [33]. The synthesized silver and copper nanoparticles covered with biomolecules are well dispersed in solutions and fairly stable up to 3 months as indicated by retention of brown colour of the solution. In case of copper nanoparticles, these results clearly indicate the formation of mixture of CuO and Cu_2_O nanoparticles as previously reported in literature [32, 34].

### 3.2. Fourier Transform Infrared (FTIR)

Fourier transform infrared (FTIR) spectrum of the experimental samples revealed two types of vibrations as stretching and bending in the wavelength range of 4000–450 cm^−1^. FTIR spectrum measurements were demonstrated to identify the major functional groups present in green alga *B. braunii* to examine their possible involvement in the synthesis of copper and silver nanoparticles. Different peaks positioned at 3435.88, 2923.49, 2852.33, 2092.30, 1637.82, 1559.61, 1414.42, 1384.79, 1069.01, 1056.17, 837.53, 781.32, 714.25, 695.06, 657, 618.16, and 532.74 cm^−1^ in FTIR spectrum of algal extract of green alga *B. braunii*. The peak at 3435.88 cm^−1^ is due to N-H and O-H stretching vibrations [35]. 2923.49 and 2852.33 cm^−1^ bands arose due to asymmetrical C-H stretching vibrations of -CH_2_ and -CH_3_ [36]. The IR peak at 2092.30 cm^−1^ is due to the alkynes groups present in the lipids and nitrile group (-CN) present in proteins of algae [37]. The 1637.82 cm^−1^ peak is a characteristic of N-H bending vibrations in amide of protein as a capping agent [38]. The peak at 1559.61 cm^−1^ showed the presence of the carboxyl group and the weak band at 1414.42 and 618.16 cm^−1^ due to COO^−^ in the amino acid residue of protein [36] [39]. The peak observed around 1384.79 cm^−1^ can be assigned to C-N stretching vibrations of amine. C-H bending vibrations by carbohydrates (glucose residue by the C-OH bond) showed the peak at 1037.17 cm^−1^. 873.53, 781.32, 695.06, and 532.74 cm^−1^ bands were demonstrated due to O-C=O bending vibrations of CO_3_^−2^, C-H rocking of lipids, and N-H wagging of amine and alkyl halide, respectively. The results of the present study have shown that the hydroxyl groups have strong ability to interact with nanoparticles. The main peaks existing in the spectrum of alga are also present in the spectra of synthesized silver and copper nanoparticles with lower intensities and slight shift. Therefore, it may be evidenced that proteins, polysaccharides, amides, and long-chain fatty acids are responsible biomolecules for bioreduction, capping, and stabilizing agents.

### 3.3. Scanning Electron Microscopy (SEM)

**Figure 3:**
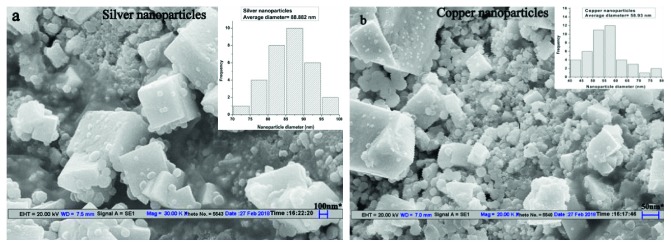
Scanning electron microscopy (SEM) images of green-synthesized (a) silver nanoparticles and (b) copper nanoparticles.

The shape and size of both biogenically synthesized nanoparticles were elucidated with the help of scanning electron microscopy (SEM) (Figures 3 and 3. The cubical, spherical, and truncated triangular shapes of silver nanoparticles were observed from scanning electron microscopy images. Copper nanoparticles observed from SEM are cubical and spherical with an elongated shape. The size of algal synthesized silver and copper nanoparticles was found to be in range of 40–100 nm and 10–70 nm, respectively. These results of SEM were confirmed by previously reported literature [14, 25].

### 3.4. X-Ray Diffraction

**Figure 4:**
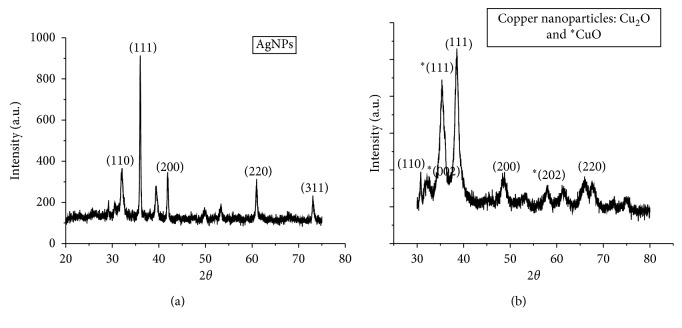
XRD patterns of biogenically synthesized (a) silver nanoparticles and (b) copper nanoparticles.

**Figure 5:**
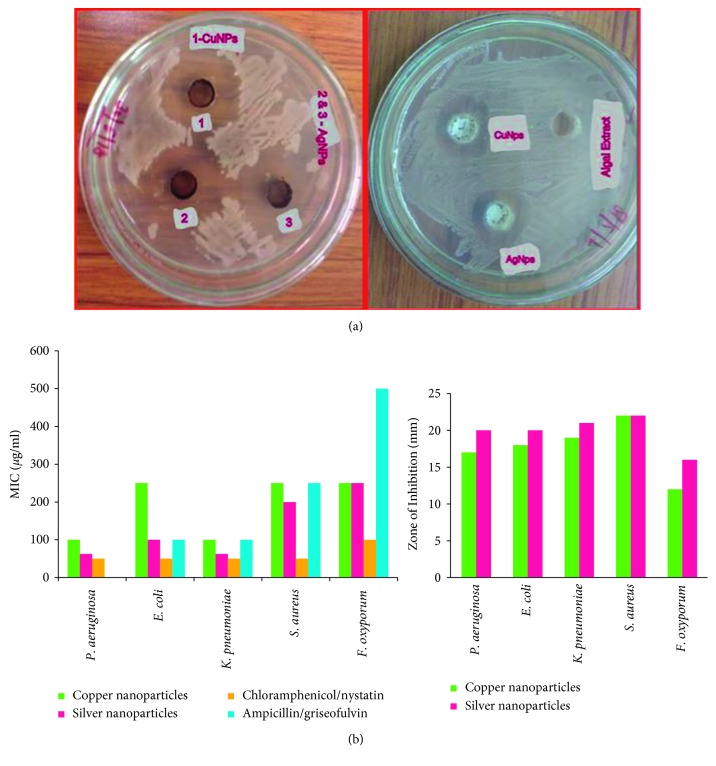
(a) Antibacterial assay: zone of inhibition against *Staphylococcus aureus* and *Pseudomonas aeruginosa*. (b) MIC value and zone of inhibition against microbes.

Furthermore, evidence for biosynthesis of silver and copper nanoparticles was confirmed by the X-ray diffraction (XRD) analysis (Figure 4). The X-ray diffraction (XRD) analysis explained the structure of silver and copper nanoparticles. In Figure 5(a), XRD patterns for silver nanoparticles between 2*θ* of 20–80° are shown. It is indexed by JCPDS card no (04–0783). XRD patterns show the diffraction peaks of silver nanoparticles at 2*θ* values for 33°, 36°, 42°, 62°, and 73° which matched with 110, 111, 200, 220, and 311 lattice planes of face-centred cubic structure, respectively [35, 40]. XRD confirmed that silver nanoparticles are crystalline in nature with the face-centred cubic structure. Average crystalline size calculated by the Debye Scherer equation is 89 nm. Furthermore, algal-synthesized copper nanoparticles in Figure 5(b) demonstrate the major XRD peaks at 30.8°, 38.66°, 48.66°, and 68.26° diffracted from the (110), (111), (200), and (220) planes of the face-centred cubic structure, respectively. It is indexed by JCPDS card no. (71–4610). In addition, three other peaks were also found at 2*θ* of 32.3°, 35.4°, and 58.1 °can be assigned to (002), (111), and (202) indicating the presence of the oxide shell around the copper nanoparticles. These peaks of the CuO phase were reported in previous findings [14, 41]. The average size of synthesized copper nanoparticles was calculated by using the Debye Scherer equation found to be around 58 nm which is correlated with that obtained from SEM images.

### 3.5. Antimicrobial Activity of Synthesized Silver and Copper Nanoparticles

Biological activity of algal extract and synthesized copper and silver nanoparticles [42, 43] was done against both bacteria (Gram positive and Gram negative) and fungus using the agar well diffusion method [44, 45] The well filled with algal extract did not show any zone of inhibition, but the nanoparticles synthesized from that algal culture showed both antibacterial and antifungal activity.

The synthesized nanoparticles showed the inhibition zone against all of the test organisms (Table 1). Maximum zone of inhibition was found against S*taphylococcus aureus* (22 mm). The next maximum zone of inhibition was recorded in *Klebsiella pneumoniae* (20 mm) followed by *Escherichia coli* (18 mm) and *Pseudomonas aeruginosa* (17 mm), and the minimum zone of inhibition was recorded against *Fusarium oxysporum* (12 mm). Furthermore, the nanoparticles synthesized by the green route are found to be highly effective against Gram-negative bacteria. However, the minimum inhibitory concentration [46] required to inhibit the growth of the microbes is less in the case of silver as compared with copper (Figure 5). These synthesized nanoparticles show least activity towards tested fungus *Fusarium oxysporum*. Even biogenically synthesized silver nanoparticles showed a lower MIC value than the positive control drug chloramphenicol against both Gram-positive and Gram-negative bacteria. Nystatin and griseofulvin were used as the positive control for fungus *Fusarium oxysporum*.

## 4. Conclusion

This present work demonstrated an ecofriendly and convenient method used for synthesis of silver and copper nanoparticles using green alga *Botryococcus braunii*. Aqueous extract of green alga can reduce silver and copper ions into silver and copper nanoparticles and has the potential to stabilise them. Biogenically synthesized nanoparticles were characterised by different techniques such as UV-visible spectroscopy, Fourier transform infrared spectroscopy, scanning electron microscopy, and X-ray diffraction and exhibited antibacterial and antifungal activity. The biogenic synthesis of metal nanoparticles can be a promising process for production of other metal and metal oxide nanoparticles which can have environmental, valuable, pharmaceutical, medical, and biotechnological applications.

## Figures and Tables

**Table 1:** Antibacterial and antifungal activity of biogenically synthesized copper and silver nanoparticles.

Nanoparticles (500 *µ*g/ml)	Zone of inhibition diameter (mm)				
	Gram-negative bacterial strains		Gram-positive bacterial strains		Fungal strain
	*Pseudomonas aeruginosa*	*Escherichia coli*	*Klebsiella pneumoniae*	*Staphylococcus aureus*	*Fusarium oxysporum*
Copper	17 ± 1.56	18 ± 0.1	19 ± 0.0	22 ± 0.88	12 ± 0.58
Silver	20 ± 1.78	20 ± 0.8	21 ± 0.60	22 ± 0.75	16 ± 1.5

All values are mean ± SD					
Minimum inhibitory concentration (*µ*g/ml)					
Copper	100	250	100	250	250
Silver	62.5	100	62.5	200	250
Ampicillin	—	100	100	250	—
Chloramphenicol	50	50	50	50	—
Nystatin	—	—	—	—	100
Griseofulvin	—	—	—	—	500

## Data Availability

The data used to support the findings of this study are available from the corresponding author upon request.
